# Tracking Applications: A Factor of Mithridatism of Personal Data and Privacy in the Post-COVID-19 Era

**DOI:** 10.1017/dmp.2020.170

**Published:** 2020-05-22

**Authors:** Anastasios Apostolos, Konstantinos Apostolos

**Affiliations:** Faculty of Medicine, University of Patras, Patras, Greece; Law School, National and Kapodistrian University of Athens, Athens, Greece

During 2020, humanity is facing an extraordinary, unknown enemy, called *severe acute respiratory syndrome coronavirus 2* (*SARS-CoV-2)*. This novel coronavirus is the cause of the coronavirus disease (COVID-19) pandemic, which has resulted in millions of confirmed cases and thousands of deaths. The control of an unprecedented outbreak demands a plethora of comprehensive measures to be adopted, such as thermal scanning, tracking applications, and devices. However, the consequences of the usage of these solutions cannot be predictable a priori, especially their impact on civil rights and liberties.

Bearing this in mind, it is critical to emphasize the impact of tracking applications or devices for COVID-19 on personal data. Their purpose is to alert users about the possibility of having personal encounters with a suspected or confirmed case of the novel coronavirus.

Apple and Google have announced their collaboration for launching such an application, based on Bluetooth information exchange.^1^ Meanwhile, the Australian Government has encouraged millions of citizens to install Bluetooth-based COVIDSafe application, which records when the user comes within 1.5 meters with a suspected case.^2^ Based on relevant technology, tracking wristbands are scrutinized in numerous countries, in order to get utilized widely, after the lockdown termination.^3^

Although the exploitation of the results of tracking systems may prove to be a precious means for COVID-19 containment, we express our concern about personal data protection. Relevant restrictions are exceptionally considered legitimate, when it is necessary for reasons of public interest, such as the advocacy of public health. It is hence vital that the personal health data, extremely sensitive information of every data subject, are processed exclusively for the COVID-19 outbreak, according with the proportionality, necessity, and the other fundamental principles of the processing of personal data.^4^ Malpractice could provoke, interalia, phenomena of stigmatization and discrimination against infected individuals.

*Exceptional situations require exceptional measures* and the post-COVID-19 era will be significantly different.^5^ However, “exceptionality must not evolve into regularity”; thus, we should monitor and prevent any form of mithridatism against our civil rights and liberties, in general, after the pandemic is eventually declared under control!
